# Waist-to-Hip Ratio, Dyslipidemia, Glycemic Levels, Blood Pressure and Depressive Symptoms among Diabetic and Non-Diabetic Chinese Women: A Cross-Sectional Study

**DOI:** 10.1371/journal.pone.0109765

**Published:** 2014-10-14

**Authors:** Yu Zheng, Qihong Sun, Kang Chen, Wenhua Yan, Changyu Pan, Juming Lu, Jingtao Dou, Zhaohui Lu, Ba Jianming, Baoan Wang, Yiming Mu

**Affiliations:** 1 Department of Endocrinology, Chinese PLA General Hospital, Beijing, China; 2 School of Medicine, Nankai University, Tianjin, China; 3 Internal Medicine, Fushun Hospital of TCM, Liaoning, China; McMaster University, Canada

## Abstract

**Objectives:**

To explore the relationship between depressive symptoms and waist-to-hip ratio, dyslipidemia, glycemic levels or blood pressure among diabetic and non-diabetic Chinese women.

**Methods:**

11,908 women aged ≥40 years were enrolled in this cross-sectional study, including 2,511 with type 2 diabetes and 9,397 without. Depressive symptoms (defined as having mild-to-severe depressive symptoms) were assessed by the Patient Health Questionnaire-9 (PHQ-9) diagnostic algorithm. The prevalence and the odds ratios (ORs) with 95% confidence intervals (CIs) for having depressive symptoms were estimated using logistic regression analysis.

**Results:**

The age-adjusted prevalence of depressive symptoms was significantly higher in non-diabetic subjects with waist-to-hip ratio (WHR) ≥0.9 (8.6%, age-adjusted OR 1.51 [95% CI 1.17, 1.95]), total cholesterol (TC)>6.22 mmol/L (8.8%, 1.58 [1.16, 2.15]), and Hemoglobin A1c (HbA1c) ≥6.00 mmol/L (7.7%, 1.69 [1.34, 2.14]), while it was significantly lower in non-diabetic subjects with diastolic blood pressure (DBP) between 80 to 89 mmHg (6.2%, 0.78 [0.64, 0.95]). These relationships remained significant even after controlling for multiple factors (WHR ≥0.9: multivariable-adjusted OR 1.39 [95% CI 1.07, 1.80]; TC>6.22 mmol/L: 1.56 [1.14, 2.12]; HbA1c ≥6.00 mmol/L: 1.64 [1.30, 2.08]; DBP 80-89 mmHg: 0.78 [0.64, 0.95]). However, no significant trend between depressive symptoms and WHC, TC, HbA1c, DBP was observed in diabetic women, and no significant trend relationship between depressive symptoms and BMI, WC, TG, or SBP was observed in both non-diabetic and diabetic women. Moreover, the prevalence of depressive symptoms was significantly higher in previously-diagnosed diabetes, compared with non-diabetic subjects, while no significant differences were observed between newly-diagnosed diabetes and non-diabetic subjects.

**Conclusion:**

The present study showed a relationship between WHR, TC, HbA1c, DBP and depressive symptoms among non-diabetic women, while no significant relationship between them was observed among diabetic women, even after controlling for multiple confounding factors.

## Introduction

Depression is a mental health illness responsible for 11.8% of YLDs (years lived with disability), with a point prevalence of approximately 6% in the Chinese population [1],[2]. Women are more prone to depression and this could be explained by gender roles and norms attached to those roles [3]. Better knowledge of its risk factors is required for effective prevention of this disorder. Recently, there is an increasing interest in the relationship between depression and metabolic syndrome or its components, including abdominal obesity, dyslipidemia, hypertension and hyperglycemia. However, researches on the relationship between them have generated controversial findings. Several studies reported a positive link between depression and metabolic syndrome or its components [4]–[6], while some negative findings have been reported as well [7]–[9]. In addition, it is estimated that 20% of patients with diabetes suffer from clinically relevant depressive symptoms [10]. There are substantive data to suggest that depression is a risk factor for type 2 diabetes, while diabetes is also a risk factor for depression, and a bidirectional relationship is formed between depression and type 2 diabetes over time [11]. The present cross-sectional study examined the relationship between depressive symptoms and type 2 diabetes. As type 2 diabetes has a marked impact on depression, the present study then divided the subjects into diabetic and non-diabetic groups, and evaluated the relationship between depression and metabolic syndrome components—abdominal obesity, dyslipidemia, and glycemic control, blood pressure–in both diabetic and non-diabetic Chinese women. Moreover, in most prior studies, the estimation of obesity only used Body mass index (BMI) or waist circumference (WC), which is easily distorted by muscle mass and bone structure or takes no account of body composition. In the present study, we examined the relationships between BMI, WC vis-a-vis waist-hip ratio (WHR), and depressive symptoms in Chinese women, as WHR is a measure of body shape and to some extent of lower trunk adiposity since it does not account for the differing ratios of adipose to lean tissue, nor does it distinguish between general or central obesity.

## Materials and Methods

### Participants and Measures

The present study was nested in an ongoing longitudinal (REACTION) study that was designed to investigate the relationship between type 2 diabetes, pre-diabetes and the risk of cancer in the Chinese population, described previously [12]. All permanent residents of Jingding, Laoshan and Gucheng communities of Beijing, China, aged ≥40 years, were invited to participate in a screening examination for diabetes. These research centers are all urban areas, with upper-middle degree of urbanization and economic development status, and the subjects represent the general middle-aged and elderly population of Beijing. Recruitment was performed by local resident associations, and was conducted in the primary health care centers located at these communities. During the recruitment period, the opportunity to participate in the study was offered daily to all residents. A total of 19,314 persons were registered between March and December, 2012. Participants meeting the following criteria were excluded: 1) those who were type 1 or other types of diabetes (n  = 205); 2) those without complete data to define depressive symptoms or diabetes (n  = 689); 3) males (n  = 6,512). A total of 11,908 females (2,511 with type 2 diabetes, 9,397 without) were included in the final analysis. We tested fasting plasma glucose (FPG) and 2 h post-load plasma glucose (PPG), and classified the participants: non-diabetic subjects, defined as an FPG level less than 7.0 mmol/L (126 mg/dL), a PPG level less than 11.1 mmol/L (200 mg/dL) and no history of diabetes; diabetes subjects, defined as an FPG level of 7.0 mmol/L or greater or a PPG level 11.1 mmol/L or greater or a history of diabetes. The two groups were then divided into two subgroups respectively according to their depressive symptoms (defined as having mild-to-severe depressive symptoms): 756 subjects with depressive symptoms, 8,705 subjects without in the non-diabetic group; 228 subjects with depressive symptoms, 2,283 subjects without in the diabetic group.

All participants received comprehensive examinations that included a detailed questionnaire, anthropometric measurement, a standard 75 g oral glucose tolerance test, and blood collection.

The self-administered questionnaire covered history of diabetes, alcohol intake, smoking habits, vocation, educational status, physical activity level, and depressive symptoms. Alcohol intake was classified as either consumption nearly/more than once a week currently or not. Smoking habits was defined as either smoking more than once a day or not. Vocation was classified as farmer or worker, official or soldier or technician, businessman, unemployed, retired and others. Educational status was classified as <high school diploma, high school graduate, and> high school diploma. Regular exercise was defined as engaging in sports more than once a week. Depressive symptoms were assessed by using the Patient Health Questionnaire-9 (PHQ-9) diagnostic algorithm, which has been described in detail elsewhere [13]. Specifically, participants were asked about how often over the last 2 weeks they had experienced each of the following symptoms: 1) little interest or pleasure in doing things; 2) feeling down, depressed, or hopeless; 3) trouble falling or staying asleep or sleeping too much; 4) feeling tired or having little energy; 5) having a poor appetite or overeating; 6) feeling bad as a failure or having let themselves or their family down; 7) having trouble concentrating on things such as reading newspaper or watching TV; 8) moving or speaking so slowly that other people could have noticed, or being so fidgety or restless that they had been moving around a lot more than usual; and 9) thinking of suicide or self-injury in some way. The responses to each item were scored as 0 point for “not at all”, 1 point for “having the symptoms for several days”, 2 points for “having the symptoms for more than half the days”, and 3 points for “having the symptoms for nearly every day”. The total score for each item produced a total depression severity score, and subjects who scored 5 or more points were defined as having mild-to-severe depressive symptoms [13]. The PHQ-9 was developed in 1999 and was derived from the Primary Care Evaluation of Mental Disorders (PRIME-MD). It has been translated into more than 80 languages and is widely used throughout the world. Compared with other instruments for screening depression, the PHQ-9 satisfies five practical considerations, which include brevity, self-administered, multipurpose, in the public domain, and easy to score. Four PHQ-9 items related to problems with sleep, fatigability, appetite, and psychomotor agitation/retardation were classified as somatic-affective symptoms, whereas 5 items, related to lack of interest, depressed mood, negative feelings about self, concentration problems and suicidal ideation, were classified as cognitive-affective symptoms of depression. It has demonstrated adequate reliability and validity and has been used in different populations, including Chinese.

Data on anthropometric measurements were collected by trained health technicians. BMI was calculated as body weight in kilograms divided by body height in meters squared (kg/m^2^). WC was measured midway between the lowest rib and the superior border of iliac crest at the end of normal expiration with a stretch-resistant measuring tape, and hip circumference (HC) was measured around the widest portion of the buttocks, with the tape parallel to the floor [14]. WHR was calculated as WC in centimeters (cm) divided by HC in cm. Blood pressure was measured three times after the subject had rested for at least 5 minutes prior to measurement, and the mean of the three measurements was used for the analysis.

Blood samples were collected by venipuncture. All participants received an oral glucose tolerance test, and were told to fast for at least 10 h before the test. Plasma glucose, serum triglycerides (TG), total cholesterol (TC), HDL cholesterol (HDL-C), and LDL cholesterol (LDL-C) were measured using an autoanalyser (Cobas 8000 modular analyzer series, Roche Diagnostics, Basel, Switzerland). Hemoglobin A1c (HbA1c) was determined by high-performance liquid chromatography using the VARIANT II Hemoglobin Testing System (Tosoh Corporation, Tokyo, Japan).

### Statistical Analysis

Statistical analysis was performed using SPSS software, version 19.0 (IBM, Chicago, IL). All continuous variables were presented as mean values and standard deviation (SD). The two-sample t-test was used to compare the differences in baseline characteristics for all continuous variables between the groups with depressive symptoms and the group without, including age, BMI, WC, WHR, TG, TC, LDL-C, HDL-C, FPG, PPG, HbA1c, systolic blood pressure (SBP), and diastolic blood pressure (DBP). Chi-square test was used for discrete data (alcohol intake, smoking habits, vocation, educational status, and exercise habits). The prevalence of depressive symptoms in each subgroup was standardized for age distribution of the sixth census of population in China by the direct method using 10-year age groupings. The odds ratios (ORs) with 95% confidence intervals (CIs) for depressive symptoms were estimated by conducting logistic regression analyses to test associations between depressive symptoms and BMI, WC, WHR, TG, TC, LDL-C, HDL-C, FPG, PPG, SBP, DBP (all used as continuous variables). First, univariate regression analysis was performed. Second, age was adjusted for (age-adjusted means and 95% CIs). Finally, multiple factors were considered, including age, alcohol intake, smoking habits, vocation, educational status, and exercise habits in the non-diabetic group; family histories of diabetes, various therapies, and duration of diabetes were also considered in the diabetic group, using a forced entry method (multivariable-adjusted means and 95% CIs). In all analyses, P <0.05 was considered to be statistically significant.

### Ethics Statement

The present study was nested in an ongoing longitudinal (REACTION) study, which is sponsored by the Chinese Society of Endocrinology and led by Rui-Jin Hospital affiliated to School of Medicine, Shanghai Jiao Tong University. All procedures used in this study were in accordance with institutional guidelines. The Committee on Human Research at Rui-Jin Hospital affiliated to School of Medicine, Shanghai Jiao Tong University approved the study protocol, and all study participants provided written informed consents [12].

## Results

The baseline characteristics of subjects with and without depression are shown in Table 1. Subjects for analysis were 11,908 females (2,511 with type 2 diabetes, 9,397 without) aged 40–93 years. The prevalence of depressive symptoms (PHQ ≥5) was 8.2%. The socio-demographic characteristics of non-diabetic subjects differed significantly by depressive symptom status except for age, vocation and educational status. The percentages of non-diabetic women who were alcohol drinker or smoker were significantly higher whereas the percentages of adults who exercised regularly were significantly lower among participants with depressive symptoms than among those without (P <0.05 for all comparisons). Non-diabetic subjects with depression had significantly higher values for WHC, TC, HDL-C, FPG and HbA1c compared to those without (P <0.05), while the mean BMI, WC, LDL-C, TG, PPG and SBP among non-diabetic participants with depressive symptoms was not significantly different from those without (all P>0.05). On the other hand, no significant difference between depressive symptoms and BMI, WC, WHC, TC, LDL-C, HDL-C, TG, FPG, PPG, or HbA1c was observed in diabetic women (all P>0.05).

**Table 1:** General characteristics of study participants with and without depressive symptoms.

	Non-diabetic				Diabetic			
	Total	With depression	Without depression	P	Total	With depression	Without depression	P
**Number**	9397	752	8645		2511	228	2283	
**Age, year**	56.4±7.6	56.8±7.3	56.4±7.6	0.092	60.2±8.5	60.7±8.7	60.2±8.5	0.380
**BMI, kg/m^2^**	25.4±3.6	25.4±3.6	25.4±3.6	0.953	26.7±3.8	26.7±3.6	26.6±3.8	0.704
**WC, cm**	81.1±8.6	80.9±8.5	81.2±8.6	0.412	85.3±9.0	84.6±8.7	85.3±9.0	0.239
**WHC**	0.86±0.06	0.87±0.06	0.86±0.06	<0.001	0.90±0.06	0.90±0.06	0.90±0.06	0.906
**Vocation, %**				0.225				0.462
farmer or worker	7.2	5.7	7.4		3.7	2.2	3.8	
official or soldier or technician	3.9	2.9	3.9		1.2	0.9	1.2	
businessman	0.5	0.3	0.5		0.2	0.0	0.2	
unemployed	4.9	5.5	4.8		4.5	2.6	4.7	
retired	82.7	84.4	82.5		89.8	93.4	89.4	
others	0.9	1.2	0.9		0.7	0.9	0.7	
**Educational status, %**				0.319				0.970
<high school diploma	38.1	40.7	37.9		52.6	53.1	52.5	
high school diploma	46.7	44.7	46.9		37.8	37.7	37.8	
> high school diploma	15.2	14.6	15.2		9.6	9.2	9.7	
**Current alcohol consumption, %**	1.3	2.8	1.2	<0.001	1.0	1.8	1.0	0.261
**Current smoker, %**	2.1	3.6	2	0.004	2.0	2.2	2.0	0.821
**Regular exercise, %**	6.8	1.6	7.2	<0.001	4.9	1.8	5.3	0.020
**History of diabetes, years**	–	–	–	–	4.1±6.5	5.2±7.4	4.0±6.4	0.009
**Family history of diabetes, %**	–	–	–	–	38.5	44.1	37.9	0.071
**Hypoglycemic agent use, %**	–	–	–	–	38.7	43.4	38.3	0.133
**Insulin use, %**	–	–	–	–	11.5	15.6	11.1	0.045
**FPG, mmol/L**	5.35±0.51	5.40±0.52	5.34±0.50	0.004	7.80±2.49	7.78±2.52	7.80±2.50	0.894
**PPG, mmol/L**	6.94±1.58	6.93±1.59	6.94±1.58	0.937	13.70±4.32	13.60±4.41	13.70±4.31	0.742
**HbA_1c_, %**	5.78±0.47	5.84±0.43	5.77±0.47	<0.001	7.15±1.48	7.17±1.40	7.15±1.49	0.894
**TC, mmol/L**	5.33±0.98	5.41±1.02	5.32±0.98	0.013	5.37±1.07	5.44±1.16	5.36±1.07	0.310
**LDL-C, mmol/L**	3.26±0.83	3.29±0.87	3.26±0.82	0.290	3.30±0.88	3.33±0.94	3.30±0.87	0.618
**HDL-C, mmol/L**	1.51±0.42	1.54±0.38	1.50±0.42	0.046	1.40±0.34	1.43±0.40	1.40±0.34	0.205
**TG, mmol/L**	1.52±1.02	1.48±0.95	1.52±1.03	0.311	1.74±1.20	1.77±0.96	1.74±1.22	0.703
**SBP, mmHg**	128.5±16.2	128.0±16.5	128.5±16.2	0.410	136.7±17.3	137.7±16.8	136.6±17.4	0.351
**DBP, mmHg**	74.1±9.3	73.0±9.5	74.2±9.2	<0.001	74.6±10.0	74.2±10.2	74.6±10.0	0.563

In Table 2, the incidence rates of depressive symptoms were 8.0% for non-diabetic subjects, 8.0% for newly-diagnosed diabetes, and 10.1% for previously-diagnosed diabetes. Compared with non-diabetic subjects, the unadjusted ORs of developing depressive symptoms were 1.00 [95% CI 0.81, 1.25] for newly-diagnosed diabetes, and 1.29 [95% CI 1.06, 1.57] for previously-diagnosed diabetes. The age-adjusted prevalence of depressive symptoms was also significantly higher in previously-diagnosed diabetes (8.9%, age-adjusted OR 1.25 [95% CI 1.02, 1.53]). These relationships remained significant even after controlling for age, alcohol intake, smoking habits, vocation, educational status, and exercise habits (multivariable-adjusted OR 1.23 [95% CI 1.01, 1.50]).

**Table 2:** Relationship between diabetes and depressive symptoms in women.

	Unadjusted			Age-adjusted			Multivariable-adjusted*	
	Prevalence (%)	OR (95%CI)	P	Prevalence (%)	OR (95%CI)	P	OR (95%CI)	P
**Non-diabetes (n = 9397)**	8.0	Reference		7.4	Reference		Reference	
**Newly-diagnosed diabetes (n = 1235)**	8.0	1.00 (0.81, 1.25)	0.987	7.6	0.98 (0.79, 1.22)	0.848	0.96 (0.77, 1.20)	0.710
**Previously-diagnosed diabetes (n = 1276)**	10.1	1.29 (1.06, 1.57)	0.010	8.9	1.25 (1.02, 1.53)	0.030	1.23 (1.01, 1.50)	0.044

*Adjusted for age, alcohol intake, smoking habits, vocation, educational status, and exercise habits.

Table 3 shows the unadjusted and age-adjusted prevalence and ORs for depressive symptoms according to the classification of WHC, TC and HbA1c among non-diabetic women. The unadjusted prevalence of depressive symptoms was significantly higher in subjects with WHR ≥0.9 (9.9%, unadjusted OR 1.54 [95% CI 1.19, 1.98]), TC>6.22 mmol/L (9.6% unadjusted OR 1.61 [95% CI 1.12, 2.19]), or HbA1c ≥6.00 mmol/L (9.1%, unadjusted OR 1.72 [95% CI 1.36, 2.16]). The age-adjusted prevalence of depressive symptoms was also higher in subjects with WHR ≥0.9 (8.6%, age-adjusted OR 1.51 [95% CI 1.17, 1.95]), TC>6.22 mmol/L (8.8%, age-adjusted OR 1.58 [95% CI 1.16, 2.15]), or HbA1c ≥6.00 mmol/L (7.7%, unadjusted OR 1.69 [95% CI 1.34, 2.14]). These relationships remained significant even after controlling for age, alcohol intake, smoking habits, vocation, educational status, and exercise habits (WHR ≥0.9: multivariable-adjusted OR 1.39 [95% CI 1.07, 1.80], TC>6.22 mmol/L: 1.56 [95% CI 1.14, 2.12]), HbA1c ≥6.00 mmol/L: 1.64 [95% CI 1.30, 2.08]. However, no significant trend relationship between depressive symptoms and BMI, WC, TG, or SBP was observed, and the relationship was not “U-shaped” from low to high categories either, among non-diabetic women, (all P>0.05, as shown in Table 3). The relationships remained insignificant after controlling for age or multiple confounding variables. The unadjusted and age-adjusted prevalence of depressive symptoms was significantly lower in subjects with DBP between 80 to 89 mmHg.

**Table 3:** Relationships between metabolic syndrome components and depressive symptoms in non-diabetic women.

	Unadjusted			Age-adjusted			Multivariable-adjusted*	
	Prevalence (%)	OR (95%CI)	P	Prevalence (%)	OR (95%CI)	P	OR (95%CI)	P
**BMI**								
<18.5 (n = 120)	9.2	1.13 (0.60, 2.12)	0.418	10.0	1.10 (0.59, 2.07)	0.368	1.11 (0.59, 2.10)	0.741
18.5–24.9 (n = 4457)	8.2	Reference		7.3	Reference		Reference	
25.0–29.9 (n = 3892)	7.7	0.94 (0.80, 1.10)	0.993	7.1	0.93 (0.79, 1.09)	0.923	0.91 (0.77, 1.07)	0.246
≥30 (n = 927)	8.2	1.00 (0.77, 1.30)	0.701	7.3	0.99 (0.76, 1.28)	0.765	0.95 (0.73, 1.23)	0.690
**WC**								
<80 cm (n = 4023)	8.4	Reference		7.5	Reference		Reference	
80–87.9 cm (n = 3368)	7.9	0.94 (0.79, 1.11)	0.459	7.6	0.92 (0.78,1.09)	0.338	0.91 (0.77, 1.08)	0.267
≥88 cm (n = 1999)	7.4	0.87 (0.71, 1.06)	0.160	6.3	0.83 (0.68, 1.02)	0.081	0.81 (0.66, 1.00)	0.041
P value for trend		0.099			0.076		0.608	
**WHR**								
<0.80 (n = 1314)	6.7	1.00		6.9	1.00		1.00	
0.80–0.85 (n = 2525)	7.2	1.08 (0.83, 1.40)	0.587	6.7	1.07 (0.82, 1.40)	0.607	1.04 (0.80, 1.35)	0.779
0.86–0.89 (n = 3000)	7.7	1.16 (0.90, 1.49)	0.262	7.1	1.15 (0.89, 1.48)	0.293	1.09 (0.84, 1.41)	0.513
≥0.9 (n = 2550)	9.9	1.54 (1.19, 1.98)	0.001	8.6	1.51 (1.17, 1.95)	0.002	1.39 (1.07, 1.80)	0.014
P value for trend		<0.001			<0.001		0.004	
**TC**								
≤4.14 mmol/L (n = 986)	6.2	1.00		5.7	1.00		1.00	
4.15–5.18 mmol/L (n = 3351)	8.3	1.37 (1.03, 1.83)	0.031	7.8	1.37 (1.03, 1.82)	0.032	1.37 (1.02, 1.82)	0.034
5.19–6.22 mmol/L (n = 3451)	7.5	1.24 (0.93, 1.65)	0.150	6.4	1.22 (0.92, 1.63)	0.173	1.20 (0.90, 1.60)	0.222
>6.22 mmol/L (n = 1598)	9.6	1.61 (1.12, 2.19)	0.003	8.8	1.58 (1.16, 2.15)	0.004	1.56 (1.14, 2.12)	0.005
P value for trend		0.028			0.043		0.064	
**LDL-c**								
<3.37. mmol/L (n = 5393)	8.0	Reference		7.4	Reference		Reference	
3.37–4.13 mmol/L (n = 2714)	7.6	0.94 (0.79, 1.12)	0.489	6.3	0.93 (0.78, 1.10)	0.393	0.92 (0.77, 1.09)	0.321
≥4.14 mmol/L (n = 1272)	8.8	1.11 (0.89, 1.38)	0.364	7.9	1.09 (0.87, 1.35)	0.466	1.08 (0.87, 1.35)	0.488
P value for trend		0.635			0.787		0.850	
**HDL-c**								
<1.03 mmol/L (n = 682)	6.5	Reference		6.1	Reference		Reference	
1.03–1.55 mmol/L (n = 4861)	7.8	1.22 (0.88, 1.68)	0.229	6.9	1.22 (0.88, 1.69)	0.228	1.22 (0.88, 1.68)	0.237
≥1.55 mmol/L (n = 3852)	8.6	1.36 (0.98, 1.89)	0.062	7.9	1.37 (0.99, 1.90)	0.059	1.36 (0.98, 1.89)	0.065
P value for trend		0.037			0.033		0.038	
**TG**								
<1.70 mmol/L (n = 6693)	8.1	Reference		7.3	Reference		Reference	
1.70–2.82 mmol/L (n = 2033)	7.6	0.93 (0.77, 1.12)	0.451	6.9	0.93 (0.77, 1.12)	0.411	0.93 (0.77, 1.12)	0.419
≥2.83 mmol/L (n = 665)	7.8	0.96 (0.71, 1.29)	0.771	6.6	0.95 (0.70, 1.27)	0.721	0.95 (0.70, 1.28)	0.722
P value for trend		0.960			0.955		0.471	
**HbA1c**								
<5.50 mmol/L (n = 1999)	5.5	1.00		5.0	1.00		1.00	
5.50–5.74 mmol/L (n = 2662)	8.0	1.49 (1.17, 1.89)	0.001	7.3	1.48 (1.16, 1.87)	0.001	1.48 (1.16, 1.87)	0.001
5.75–5.99 mmol/L (n = 1865)	9.1	1.71 (1.33, 2.20)	<0.001	8.8	1.69 (1.32, 2.18)	<0.001	1.65 (1.28, 2.12)	<0.001
≥6.00 mmol/L (n = 2871)	9.1	1.72 (1.36, 2.16)	<0.001	7.7	1.69 (1.34, 2.14)	<0.001	1.64 (1.30, 2.08)	<0.001
P value for trend		<0.001			<0.001		<0.001	
**FPG**								
<6.1 mmol/L (n = 8572)	7.9	Reference		7.1	Reference		Reference	
6.1–6.9 mmol/L (n = 825)	8.8	1.13 (0.88, 1.45)	0.349	9.6	1.11 (0.86, 1.43)	0.418	1.07 (0.83, 1.38)	0.620
**PPG**								
<7.8 mmol/L (n = 6848)	8.0	Reference		7.4	Reference		Reference	
7.8–11.0 mmol/L (n = 2532)	7.9	0.99 (0.83, 1.17)	0.883	6.8	0.96 (0.81, 1.14)	0.668	0.95 (0.80, 1.13)	0.558
**SBP**								
<120 mmHg (n = 2982)	8.2	Reference		7.3	Reference		Reference	
120–139 mmHg (n = 4323)	8.2	1.01 (0.85, 1.19)	0.936	7.8	0.98 (0.82, 1.16)	0.791	0.96 (0.81, 1.14)	0.645
≥140 mmHg (n = 2071)	7.3	0.89 (0.72, 1.10)	0.273	6.9	0.83 (0.67, 1.04)	0.105	0.82 (0.65, 1.02)	0.072
P value for trend		0.323			0.127		0.085	
**DBP**								
<80 mmHg (n = 6974)	8.5	Reference		7.6	Reference		Reference	
80–89 mmHg (n = 1963)	6.7	0.77 (0.64, 0.94)	0.010	6.2	0.78 (0.64, 0.95)	0.012	0.78 (0.64, 0.95)	0.013
≥90 mmHg (n = 462)	7.1	0.83 (0.58, 1.20)	0.322	5.8	0.85 (0.59, 1.22)	0.364	0.85 (0.59, 1.22)	0.371
P value for trend		0.018			0.024		0.025	

*Adjusted for age, alcohol intake, smoking habits, vocation, educational status, and exercise habits.

Table 4 shows the unadjusted and age-adjusted prevalence and ORs for depressive symptoms according to the classification of WHC, TC, HbA1c, DBP, BMI, WC, TG and SBP among diabetic women. However, none of them was observed to be significantly associated with depressive symptoms in diabetic women (all P>0.05, as shown in Table 4).

**Table 4:** Relationships between metabolic syndrome components and depressive symptoms in diabetic women.

	Unadjusted			Age-adjusted			Multivariable-adjusted*	
	Prevalence (%)	OR (95%CI)	P	Prevalence (%)	OR (95%CI)	P	OR (95%CI)	P
**BMI**								
<18.5 (n = 17)	5.9	0.67 (0.09, 5.16)	0.704	5.8	0.67 (0.09, 5.11)	0.696	0.82 (0.11, 6.42)	0.848
18.5–24.9 (n = 837)	8.5	Reference		6.7	Reference		Reference	
25.0–29.9 (n = 1217)	9.7	1.16 (0.85, 1.58)	0.350	8.9	1.16 (0.85, 1.58)	0.345	1.19 (0.87, 1.63)	0.280
≥30 (n = 439)	8.7	1.02 (0.68, 1.54)	0.916	8.0	1.03 (0.68, 1.56)	0.880	1.10 (0.72, 1.68)	0.651
**WC**								
<80 cm (n = 621)	9.3	Reference		7.6	Reference		Reference	
80–87.9 cm (n = 942)	10.2	1.10 (0.78, 1.55)	0.581	10.0	1.09 (0.78, 1.54)	0.610 (0.771,1.522)	1.10 (0.78, 1.57)	0.584
≥88 cm (n = 946)	7.8	0.82 (0.58, 1.18)	0.291	7.3	0.81 (0.57, 1.17)	0.259 (0.534,1.173)	0.82 (0.57, 1.19)	0.300
P value for trend		0.228			0.201		0.733	
**WHR**								
<0.80 (n = 127)	11.0	Reference		8.8	Reference		Reference	
0.80–0.85 (n = 386)	7.3	0.63 (0.32, 1.24)	0.182	6.4	0.63 (0.32, 1.24)	0.178	0.68 (0.34, 1.36)	0.274
0.86–0.89 (n = 791)	9.1	0.81 (0.44, 1.48)	0.491	7.8	0.80 (0.44, 1.47)	0.468	0.89 (0.47, 1.67)	0.719
≥0.9 (n = 1205)	9.5	0.84 (0.47, 1.52)	0.570	9.4	0.82 (0.46, 1.49)	0.519	0.86 (0.46, 1.60)	0.633
P value for trend		0.602			0.690		0.740	
**TC**								
≤4.14 mmol/L (n = 293)	10.2	Reference		10.3	Reference		Reference	
4.15–5.18 mmol/L (n = 837)	7.6	0.73 (0.46, 1.15)	0.168	7.0	0.73 (0.46, 1.15)	0.177	0.73 (0.46, 1.17)	0.188
5.19–6.22 mmol/L (n = 887)	8.6	0.82 (0.53, 1.28)	0.386	7.0	0.82 (0.53, 1.29)	0.392	0.87 (0.55, 1.37)	0.549
>6.22 mmol/L (n = 491)	11.8	1.17 (0.74, 1.87)	0.500	12.3	1.18 (0.74, 1.88)	0.491	1.28 (0.79, 2.07)	0.318
P value for trend		0.161			0.161		0.069	
**LDL-c**								
<3.37. mmol/L (n = 1356)	8.8	Reference		7.9	Reference		Reference	
3.37–4.13 mmol/L (n = 733)	9.1	1.05 (0.76, 1.43)	0.780	7.7	1.05 (0.76, 1.43)	0.778	1.13 (0.82, 1.56)	0.452
≥4.14 mmol/L (n = 419)	10.0	1.16 (0.80, 1.68)	0.437	11.7	1.16 (0.80, 1.68)	0.434	1.26 (0.86, 1.83)	0.239
P value for trend		0.451			0.448		0.214	
**HDL-c**								
<1.03 mmol/L (n = 284)	8.5	Reference		7.0	Reference		Reference	
1.03–1.54 mmol/L (n = 1521)	8.6	1.02 (0.65, 1.61)	0.929	7.8	1.02 (0.65, 1.60)	0.939	1.01 (0.64, 1.60)	0.956
≥1.55 mmol/L (n = 706)	10.3	1.25 (0.77, 2.03)	0.367	9.8	1.24 (0.76, 2.01)	0.384	1.23 (0.76, 2.01)	0.399
P value for trend		0.220			0.235		0.249	
**TG**								
<1.70 mmol/L (n = 1524)	8.7	Reference		7.5	Reference		Reference	
1.70–2.82 mmol/L (n = 730)	9.0	1.04 (0.76, 1.42)	0.806	9.2	1.04 (0.77, 1.42)	0.791	1.10 (0.80, 1.50)	0.566
≥2.83 mmol/L (n = 257)	11.3	1.33 (0.87, 2.04)	0.189	8.8	1.34 (0.88, 2.05)	0.179	1.44 (0.94, 2.22)	0.098
P value for trend		0.256			0.243		0.121	
**HbA1c**								
<6.50 mmol/L (n = 983)	7.9	Reference		6.7	Reference		Reference	
6.50–7.49 mmol/L (n = 852)	10.4	1.36 (0.99, 1.88)	0.061	9.9	1.36 (0.98, 1.88)	0.064	1.24 (0.89, 1.72)	0.208
≥7.50 mmol/L (n = 721)	9.0	1.16 (0.82, 1.64)	0.412	8.3	1.16 (0.82, 1.64)	0.406	0.95 (0.65, 1.39)	0.782
P value for trend		0.358			0.352		0.885	
**FPG**								
<6.1 mmol/L (n = 547)	9.0	Reference		7.3	Reference		Reference	
6.1–6.9 mmol/L (n = 596)	9.4	1.05 (0.71, 1.58)	0.798	9.6	1.06 (0.71, 1.58)	0.782	1.06 (0.70, 1.60)	0.786
7.0–7.9 mmol/L (n = 575)	8.7	0.97 (0.64, 1.46)	0.877	8.0	0.98 (0.65, 1.48)	0.907	0.98 (0.65, 1.50)	0.938
≥8.0 mmol/L (n = 793)	9.2	1.03 (0.71, 1.51)	0.877	8.7	1.04 (0.71, 1.52)	0.849	0.88 (0.59, 1.31)	0.525
P value for trend		0.978			0.948		0.441	
**PPG**								
<11.1 mmol/L (n = 532)	10.2	Reference		11.2	Reference		Reference	
11.1–12.9 mmol/L (n = 736)	8.4	0.81 (0.56, 1.19)	0.293	7.3	0.81 (0.56, 1.19)	0.291	0.84 (0.56, 1.25)	0.382
13.0–14.9 mmol/L (n = 523)	7.8	0.75 (0.49, 1.15)	0.191	7.2	0.75 (0.49, 1.15)	0.193	0.78 (0.51, 1.20)	0.259
≥15.0 mmol/L (n = 720)	9.9	0.97 (0.67, 1.41)	0.866	8.1	0.97 (0.67, 1.41)	0.865	0.89 (0.60, 1.31)	0.545
P value for trend		0.954			0.955		0.573	
**SBP**								
<120 mmHg (n = 384)	7.8	Reference		7.3	Reference		Reference	
120–139 mmHg (n = 1146)	8.8	1.14 (0.75, 1.74)	0.544	7.7	1.13 (0.73, 1.73)	0.590	1.16 (0.75, 1.78)	0.515
≥140 mmHg (n = 973)	10.0	1.31 (0.85, 2.00)	0.220	10.2	1.27 (0.82, 1.97)	0.251	1.31 (0.84, 2.04)	0.232
P value for trend		0.184			0.243		0.207	
**DBP**								
<80 mmHg (n = 1787)	9.5	Reference		9.0	Reference		Reference	
80–89 mmHg (n = 556)	8.3	0.86 (0.61, 1.21)	0.378	7.3	0.87 (0.62, 1.23)	0.444	0.90 (0.63, 1.28)	0.550
≥90 mmHg (n = 160)	7.5	0.77 (0.42, 1.42)	0.403	7.0	0.79 (0.43, 1.46)	0.454	0.86 (0.46, 1.60)	0.637
P value for trend		0.254			0.318		0.487	

*Adjusted for age, alcohol intake, smoking habits, vocation, educational status, exercise habits, family histories of diabetes, various therapies, and duration of diabetes.

## Discussion

The prevalence of depressive symptoms (PHQ ≥5) was 8.2% in this study, which is higher than the survey in China (approximately 6%) during 2001–2005 [2]. The first reason for the higher prevalence is the age difference, the mean age of this study is 57.2 which is higher than 44.8 in the 2001–2005 survey. The second reason is this study only includes women, who are more prone to depression. Moreover, the difference may be also attributed to sample size variation and the measurement of depression.

The present cross-sectional study demonstrated a higher prevalence of depressive symptoms in non-diabetic women with higher WHR, TC and HbA1c, while no significant relationships between depressive symptoms and WHR, TC or HbA1c were observed among diabetic women, and no significant trend relationship between depressive symptoms and BMI, WC, TG, or SBP was observed in both non-diabetic and diabetic women, even after controlling for multiple confounding factors. Moreover, the prevalence of depressive symptoms was significantly higher in previously-diagnosed diabetes, compared with non-diabetic subjects, while no significant differences were observed between newly-diagnosed diabetes and non-diabetic subjects. The result suggests that mental health status should be monitored and evaluated in non-diabetic women with high WHR, TC and HbA1c, and the depressive symptoms in diabetic women are more complicated.

To our best knowledge, the present study is the first report about the relationship between WHR and depressive symptoms, which divided women into diabetic and non-diabetic groups. We found that WHR ≥0.9 was significantly associated with high prevalence and OR of depressive symptoms in non-diabetic women, whereas BMI and WC were not significantly associated with depressive symptoms. The relationship observed between WHR and depressive symptoms is probably explained by abdominal obesity.

Previous studies on the relationship between obesity and depression have generated controversial findings. Some studies have shown that high BMI was associated with lowered risk of depression [15]–[19]. However, an absent or even an inverse relationship has also been reported. For instance, some studies have shown that BMI is positively related to depression [20]–[24] and others have shown that increased WC or abdominal obesity is strongly associated with variety of mental disorders, including depression [25],[26]. A drawback of using BMI to investigate the relationship between obesity and depressive symptoms is that it fails to account for the differing ratios of adipose to lean tissue and is easily distorted by muscle mass and bone structure. A limitation of WC alone as a measure is that it takes no account of body composition, whereas WHR is a measure of body shape and to some extent of lower trunk adiposity [27]. Taken together, the present study further suggests that WHR may be a preferred predictor of depression in this population.

Serum lipids and lipoproteins are also argued to be associated with depression, but findings regarding this link have been inconsistent. Some studies have found lower total cholesterol in subjects with depressive symptoms versus controls [28]–[30], while others have reported higher total cholesterol or found no differences [31],[32]. Levels of LDL-C, HDL-C, and TG were assessed in relation to depression or depressive complaints as well, but less extensively [33],[34].

In the present study, we demonstrated a higher prevalence of depressive symptoms in non-diabetic women with TC>6.22 mmol/L, while no differences were found for LDL-C, HDL-C or TG levels. The results suggest that the high TC other than LDL-c, HDL-C or TG levels is associated with depressive symptoms in non-diabetic women.

The relationship between glycemic level and depression is still poorly understood. Congruent with prior studies, we found no evidence of a statistically significant relationship between depressive symptoms and glycemic control in type 2 diabetic women [35],[36]. However, we found that high HbA1c level was shown to be associated with depressive symptoms in non-diabetic women, and to our best knowledge, no previous study investigated the relationship between HbA1c level and depressive symptoms in non-diabetic women.

The reported findings on the relationship between blood pressure and the occurrence of depressive symptoms are also conflicting. Low blood pressure has been shown to be associated with depressive symptoms in a number of population studies of both younger and older subjects [37]–[41], although others also have reported null findings [42],[43].

In the present study, the relationship between DBP and depressive symptoms was also shown to be “U-shaped” from high to low categories, independent of other variables, while SBP was not statistically significant among non-diabetic women. Among diabetic women, neither DBP nor SBP showed significant relationship with depressive symptoms. The result suggests a complex relationship between blood pressure and depression that probably depends on age and race differences.

The mechanism of the relationship between depression and abdominal obesity, dyslipidemia and higher glycemic levels is not yet established, and the relationship between them appears to be bi-directional [44]. First, depression has been positively associated with abdominal obesity, dyslipidemia and higher glycemic levels. It can be assumed that visceral adipose tissue plays a key role in the relationship between them via a higher production of inflammatory cytokines (e.g., C-reactive protein and interleukin 6) [45],[46]. Second, depression can activate the hypothalamic-pituitary- adrenocortical (HPA) axis, producing hypersecretion of corticotrophin-releasing hormone, adrenocorticotropic hormone, and cortisol, which could affect abdominal fat accumulation, lipid and glucose metabolism [47]–[49]. The mechanism of the relationship between lower blood pressure and depressive symptoms is unclear either. It is possible that low blood pressure may lead to depressive symptoms via reduced cerebral perfusion [37]. Taken together, the potential mechanisms are complex, and more studies are needed to explore the mechanisms underlying this reciprocal relation, which will be crucial for the prevention and treatment of depression.

There are several limitations in the present study. First, the causal relationship between WHR, TC, HbA1c or DBP and depressive symptoms cannot be established based on the nature of our cross-sectional study. Second, we used PHQ-9 as a measurement rather than a clinical diagnosis to assess depressive symptoms, which was self-reported and may lead to inevitable recall bias, although the PHQ-9 depression assessment has been validated in the general population including those who are overweight and obese as well as in patients with diabetes. Third, no association between depressive symptoms and metabolic syndrome components in diabetic women was observed, which may be related to small sample size. Further large-scale studies are needed to confirm the result and to fully ascertain the mechanisms underlying the difference between non-diabetic and diabetic women. Forth, the study was performed in middle-aged and elderly Chinese, which limits its generalizability to other ages and ethnics. Finally, there was no information on history of depression and current medication for depression, and antidepressant treatments, which are associated with weight gain, were not taken into account in the present study. Moreover, the differences from other studies could also be attributed to sample size variation, population difference, and the measurement of depression.

In conclusion, the present study indicates that high WHR, TC, HbA1c are significantly associated with increased prevalence and ORs of depressive symptoms in non-diabetic women, while middle DBP is significantly associated with decreased prevalence and ORs of depressive symptoms, even after adjustment of multiple-covariates. The results of the present study have important clinical implications. Considering that high WHR and TC are common in Chinese women, the control of abdominal obesity and hypercholesterolemia may be important for alleviating mood symptoms among non-diabetic women. On the other hand, the cause of depression is more complicated in diabetic women. The prevalence of depressive symptoms was significantly higher in previously-diagnosed diabetes, compared with non-diabetic subjects, while no significant differences were observed between newly-diagnosed diabetes and non-diabetic subjects. Moreover, WHR, TC, HbA1c, DBP may be less important in diabetic women.
